# Editorial: Language, Cognition, and Gender

**DOI:** 10.3389/fpsyg.2016.00772

**Published:** 2016-05-31

**Authors:** Alan Garnham, Jane Oakhill, Lisa Von Stockhausen, Sabine Sczesny

**Affiliations:** ^1^Psychology, University of SussexBrighton, UK; ^2^Psychology, University of Duisburg-EssenEssen, Germany; ^3^Psychology, University of BernBern, Switzerland

**Keywords:** language, cognition, gender, gender-fair language, gender stereotypes

Gender inequality remains a contentious issue in many societies, despite legislative, and other less formal attempts to tackle it. It is perpetuated, in part, by gender stereotyping. Previous research indicates that language contributes to gender inequality in various ways: Gender-related information is transmitted through formal and semantic features of language, such as the grammatical category of gender, through gender-related connotations of role names (e.g., *manager, secretary*), and through customs of denoting social groups with derogatory as opposed to neutral names. Both as a formal system and as a means of communication, language passively reflects culture-specific social conditions. Furthermore, language can also be used to express actively, and can potentially perpetuate, those conditions. Tackling these issues successfully depends on a proper understanding of their cognitive and societal underpinnings, but also on understanding the effects of attempted interventions. With these points in mind, the editors of this Special Topic, in collaboration with other colleagues, proposed a *Marie Curie Initial Training Network* entitled *Language, Cognition, and Gender* (ITN LCG), to address a range of questions about language and gender inequality. This project received funding from the European Commission's Seventh Framework Programme (FP7/2007-2013). ITN LCG included 10 European universities in the Czech Republic, Germany, Italy, Norway, Spain, Switzerland, and the United Kingdom, together with 12 associate partners in Germany, Italy, Switzerland, and the United Kingdom.

The research conducted within the ITN was organized into four work packages, addressing the questions of:

- how languages shape cognitive representations of gender
- how features of European languages correspond with gender equality in European societies
- how language contributes to social behavior toward the sexes
- how gender equality can be promoted through strategies for gender-fair language use.

These questions also appeared in the call for papers for this Special Topic, as it was intended that the Special Topic should showcase findings from ITN LCG together with related research.

Reflecting ITN LCG's focus on both cognitive and broader language-based and societal issues, the Special Topic has nine papers in Frontiers in Psychology, Cognition, and eight papers in Frontiers in Psychology, Language Sciences. However, it was originally thought that all papers would be referenced in both sections, so that the allocation of a paper to either Cognitive or Language Sciences is of no particular significance. Of the nine papers in the Cognition section, seven report work from ITN LCG and the other two (Garnham and Yakovlev; Garnham et al.) report related work by Garnham and colleagues, which arose out of discussions within ITN LCG, but which was carried out by students at the University of Sussex who were not funded from the ITN LCG grant. Of the eight papers in the Language Sciences section, six report work from ITN LCG. Of the other two, one (Wolter et al.) was carried out in collaboration with members of the ITN LCG, whereas the other (Gustafsson Sendén et al.) was an independent study, closely related to the interests of ITN LCG.

In keeping with ITN LCG's multidisciplinary approach, the contributors to this Special Topic include both cognitive and social psychologists, and linguists. For the most part the contributions report original research, with a wide range of methods, from surveys to electro-physiological studies. In addition, the Special Topic includes one Review paper (Sczesny et al.) and one Hypothesis and Theory paper (Esaulova and von Stockhausen). Most of the contributions address questions about either the cognitive representation of gender or the use and effects of gender-fair language. They present a range of complementary studies, which make a substantial contribution to the understanding of these important issues.

## Cognitive representation of gender

The Special Topic includes papers from four ITN LCG laboratories with a strong interest in cognitive representations of gender (University of Duisburg-Essen, Basque Center on Cognition, Brain and Language, University of Modena and Reggio Emilia, University of Sussex). The main focus in these studies is on how gender stereotypes, other gender-biased content, and grammatical information combine to determine the representation of the gender of characters, in word as well as in text processing. In this work a particular notion of stereotyping is used, one based on people's estimates of the proportions of females and males filling certain roles. The experimental techniques include ERPs, eye tracking, and various reading time and reaction time paradigms, in particular one devised by Oakhill et al. (2005), in which people have to say whether two terms, for example uncle and nurse, can refer to the same person. The relation between stereotypes defined in this way, and real world ratios of females to males, is explored in the paper by Garnham et al.

Canal et al. showed different ERP signatures for the gender mismatch effect for definitional and stereotype-based information in the interpretation of English reflexives. They also included individual difference measures, which correlated with participants' performance. In another ERP study, Su et al. looked at the corresponding match/mismatch effect for Chinese reflexives following definitional or stereotypical role nouns. They argue that the Chinese equivalent of “himself” is the default reflexive, and requires less complex processes of resolution than the Chinese equivalent of “herself”.

Reali et al. present an eye tracking study in which people were described as performing typically female or typically male activities without using role names. Strength of typicality was manipulated, but evidence for stereotyping was found with both strong and weak typicality, suggesting a difference between typicality and stereotyping.

Siyanova-Chanturia et al. tested Italian 3rd and 5th graders, and young and older adults on the Oakhill et al. (2005) two-word task. They found some interesting asymmetries for both male vs. female participants and for masculine vs. feminine stereotyped role names. Their most important finding, however, was that the basic stereotyping effect was seen in all age groups.

Hanulíková and Carreiras looked not at stereotyping, but at gender information conveyed by a speaker's voice. The effect of this information was contrasted with that of morphosyntactic marking on subject nouns, and the study investigated how both types of information affected subject-verb agreement. Different ERP signatures were found for the two types of agreement.

Garnham and Yakovlev report a study of the reading of short passages in Russian with either singular or plural stereotyped role nouns. The grammar of Russian has complexities not seen in languages previously investigated in studies of this kind, and Garnham and Yakovlev found complex interactions between grammatical and stereotypical information about gender. In addition, they provide a set of stereotype norms for 160 role names in Russian.

The final experimental paper in this group is Finnegan et al.'s study of the use of counter-stereotype pictures to overcome automatic stereotyping. This study used the two-word task, and looked at changes in responding before and after exposure to a set of pictures with people in either stereotypical or counter-stereotyped roles.

The Hypothesis and Theory paper by Esaulova and von Stockhausen argues that gender should be treated as a prominence feature, which influences, for example, the assignment of thematic roles. Consistent with the notions of stereotyping and discrimination, stereotypically masculine role names were more easily integrated with agent roles than were stereotypically feminine ones.

## Gender fair language

Another group of papers, including work from ITN LCG labs in Berlin (Free University), Bern, and Padua, focuses specifically on the use of gender fair language. As Sczesny et al. point out in their Review, two methods of eliminating the overuse of masculine or male-related terms are neutralization (e.g. replacing policeman with police officer) and feminization [e.g. replacing an allegedly generic masculine plural, such as German Lehrer (teacher) with a word pair Lehrerinnen und Lehrer]. These authors look at the use of these two strategies and how they can feed into future research and policy making.

An example of research of this kind is presented by Horvath et al. who show that word pairs such as Lehrerinnen und Lehrer increase female visibility in occupations, but decrease estimated salaries (though not competence/status), though for female-biased professions only. Similarly, in a study of Swiss French speaking adolescents, Vervecken et al. found that the use of word pairs reduces differences in ascription of success in occupations and ascriptions of warmth. Ascriptions of competence were not affected by language forms. Hansen et al. looked at the effect of including generic masculine or word pairs in German newspaper reports. The linguistic forms used in the reports affected readers own use of word pairs, and led to more gender-balanced representations.

Gustafsson Sendén et al. report on a different attempt to use gender-fair language, the introduction, in Sweden in 2012, of a new gender neutral pronoun, “hen.” Initial hostile attitudes to the new pronoun reduced dramatically over the following 4 years, though take up of use of the word was relatively slow. From a broader perspective, Formanowicz et al. review the effects of gender fair language on support for social initiatives in Poland and Austria. Gender fair language is a relatively new idea in Poland and had detrimental effects on support for social initiatives. In Austria, where it is better established, it had positive effects, suggesting the need for gender fair language to establish itself in a particular society before it can be effective in reducing discrimination.

## Decision making and teachers' attitudes

In the two final studies of the Special Topic, Fabre et al. showed that female responders were typically treated less fairly in the ultimatum game, and when female responders were treated fairly, more cognitive effort was needed. Wolter et al. showed that schoolteachers' attitudes are an important factor affecting whether the stereotype “reading is for girls,” measured by both motivation to read and competence at reading, is realized.

All in all, the papers in the Special Topic both contribute to our understanding of how language determines the representation of gender and feed in to discussion of and strategies for mitigating against negative effects of language.

## Author contributions

AG, wrote draft, incorporated comments of other authors into revised version. JO, LS, SS, commented on draft. AG, JO, LS, SS, proposed and edited the Special Topic for which this is the editorial.

## Funding

This Special Topic derives from the Marie Curie Initial Training Network: Language, Cognition and Gender, funded by the European Commission's Seventh Framework Programme (FP7/2007-2013) under grant agreement n°237907 (http://www.unifr.ch/psycho/itn-lcg/en). Most, but not all, of the work in the Special Topic was funded by this Marie Curie Initial Training Network (see individual papers for acknowledgement of funding).

### Conflict of interest statement

The authors declare that the research was conducted in the absence of any commercial or financial relationships that could be construed as a potential conflict of interest.
